# Supplementary material for: The Large Molecular Weight Polysaccharide from Wild Cordyceps and Its Antitumor Activity on H22 Tumor-Bearing Mice
Source: Molecules. 2023 Apr 10;28(8):3351. doi: 10.3390/molecules28083351 (PMC10141569; doi:10.3390/molecules28083351)
Supplement: Supplementary file 1 [file molecules-28-03351-s001.zip › molecules-2319906-supplementary.pdf]

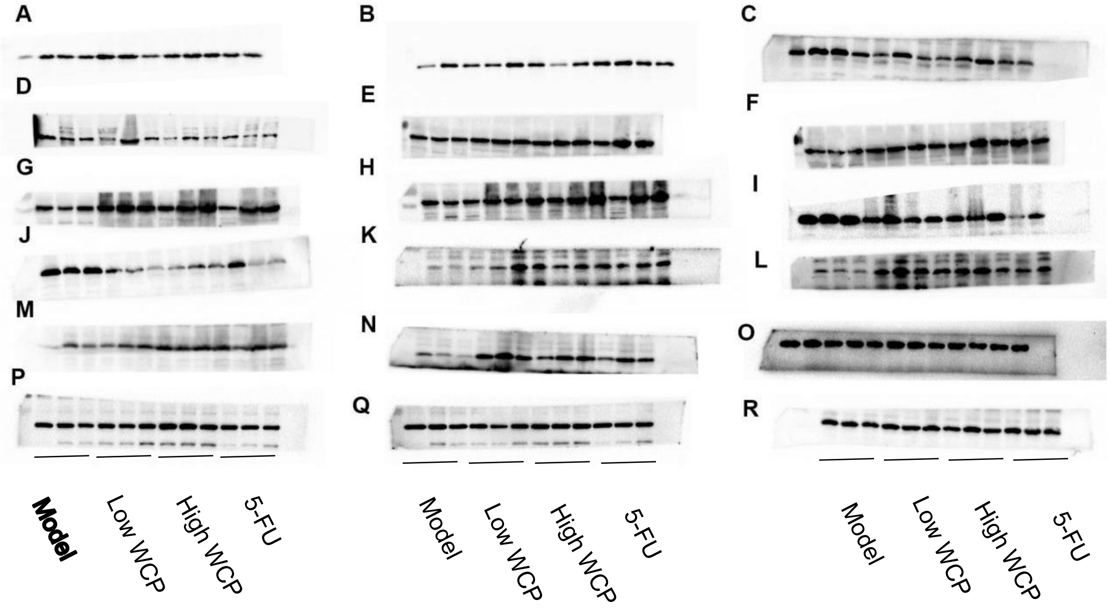

Figure S1. Images of the Western blot in tumors of H22 mice. The protein of STAT3 (A-B), p-STAT3 (C-D), Caspase8 (E-F), Caspase3 (G-H), Bcl2 (I-J), Bax (K-L), Cyto-c (M-N), β-actin (O-R).
